# Second-Line Chemotherapy for Intrahepatic Cholangiocarcinomas: What Is the Real Gain?

**DOI:** 10.3390/life13112170

**Published:** 2023-11-06

**Authors:** Ingrid Garajová, Fabio Gelsomino, Massimiliano Salati, Anna Mingozzi, Marianna Peroni, Stefania De Lorenzo, Alessandro Granito, Francesco Tovoli, Francesco Leonardi

**Affiliations:** 1 Medical Oncology Unit, University Hospital of Parma, 43126 Parma, Italy; anna.mingozzi@unipr.it (A.M.); marianna.peroni@unipr.it (M.P.); il-france@hotmail.it (F.L.); 2 Department of Oncology and Hematology, University Hospital of Modena, 41124 Modena, Italy; fabiogelsomino83@yahoo.it (F.G.); maxsalati@live.it (M.S.); 3 Oncology Unit, Azienda USL Bologna, 40127 Bologna, Italy; stefania.delorenzo@libero.it; 4 Division of Internal Medicine, Hepatobiliary and Immunoallergic Diseases, IRCCS Azienda Ospedaliero-Universitaria di Bologna, 41138 Bologna, Italy; alessandro.granito@unibo.it (A.G.); francesco.tovoli@unibo.it (F.T.)

**Keywords:** intrahepatic cholangiocarcinoma, second-line chemotherapy, prognosis

## Abstract

Background: The presence of actionable alterations in advanced biliary tract cancer patients opened new therapeutic possibilities for second-line treatments. However, for around 60% of the patients, chemotherapy remains the only therapeutic option. The aim of our study was to evaluate outcomes and prognostic parameters in patients with intrahepatic cholangiocarcinomas treated with second-line chemotherapy. Methods: A total of 255 consecutive metastatic intrahepatic cholangiocarcinoma (ICC) patients were retrospectively reviewed and clinicopathologic and survival data were collected. Results: Fourty-four percent of ICC patients underwent second-line chemotherapy. In particular, younger ICC patients with better ECOG PS status, and with disease control after first-line chemotherapy were those who were treated with second-line treatments. Median progression-free survival in the patients treated with second-line chemotherapy was 3 months. Finally, the patients affected by intrahepatic cholangiocarcinoma with better ECOG PS, with prior surgical resection of the primary tumor, who responded to first-line chemotherapy, and had better progression-free survival with second-line chemotherapy, were associated with better outcomes in multivariate analysis. Conclusions: Not all patients seem to benefit from second-line chemotherapy. To improve therapeutic decisions, performance status and disease control with first-line chemotherapy should lead to the decision on the usefulness of second-line treatments in advanced ICC patients.

## 1. Introduction

Biliary tract cancers (BTCs) are rare tumors that account for less than 1% of all cancers worldwide, even though both incidence and mortality have risen in the last decades [1,2,3,4,5]. The term BTC refers to a spectrum of invasive tumors, mainly adenocarcinomas, arising from the gallbladder/cystic duct (gallbladder carcinoma) or the biliary tree (cholangiocarcinoma, CCA). CCA are further divided into intrahepatic cholangiocarcinoma localized within the liver (ICC) or extrahepatic cholangiocarcinoma localized outside the liver (ECC) [1,2,3,4]. Surgery represents the only chance of long-term cure in BTCs but patients are rarely diagnosed with disease suitable for surgical resection. Unresectable and metastatic BTCs have a poor early stage prognosis with 5-year overall survival (OS) of less than 10% [1,2,3,4]. In advanced BTC (aBTC), the first-line standard of care has remained unchanged for more than 10 years; it was established from the results of the ABC-02 trial that reported significantly improved OS of 11.7 months with a combination of gemcitabine and cisplatin, versus 8.1 months with gemcitabine monotherapy [6]. More recently, evidence has suggested the potential role of immunotherapy in this setting. In the phase III trial, TOPAZ-1, the addition of the immune checkpoint inhibitors (ICI) durvalumab (programmed death-ligand 1 (PD-L1) blocking antibody) and cisplatin-gemcitabine led to a statistically significant improvement in both OS and progression-free survival (PFS) and should therefore be considered for standard first-line treatment [7,8,9]. The benefit of adding immunotherapy to the gemcitabine-cisplatin chemotherapy backbone in patients with advanced unresectable or metastatic BTC has been confirmed in the phase III KEYNOTE-966 trial where the addition of pembrolizumab prolonged median OS compared to placebo [10]. After progression on first-line chemotherapy, patients with advanced BTC often experience a rapid decline in performance status (PS). Only a small percentage (15–25%) are eligible to receive second-line therapy, typically younger patients and those with longer PFS following first-line chemotherapy [3,4,11]. Given the rarity of these tumors and the heterogeneity of the patient population, generally characterized by chemorefractory disease, it has been difficult to perform randomized clinical trials focused on second-line therapy, and the role of second-line chemotherapy has remained unclear for a long time. Fluoropyrimidine-based regimens have commonly been used on the grounds of modest response rates reported in multiple retrospective analyses and single-arm prospective studies [12,13]. The ABC-06 trial has been the first and, so far, the only phase 3 randomized trial exploring the role of second-line chemotherapy and demonstrated that the addition of FOLFOX to active symptoms controlled and improved median 6-month and 12-month OS with an acceptable toxicity profile [14].

Because aBTC were found to be enriched in genomic alterations, several trials have been exploring the potential role in the second-line settings of targeted therapies (i.e., FGFR2 and IDH1 inhibitors [13,15]). A significant proportion of biliary tract cancers have an actionable molecular alteration. According to some authors, ICC have the largest proportion of potentially actionable molecular alterations (approximately 50%), while the other sites of BTC demonstrated up to only 30% [16,17]. For this reason, Next Generation Sequencing of both DNA and RNA is recommended in these patients during or at the progression of first-line therapy [1,18]. The natural history of these subgroups is not yet understood and the correct timing of targeted agents, with respect to standard chemotherapy, is an area for future translational and clinical research. Although there has been significant progress, different challenges have to be faced. Some BTC patients are unlikely to be able to receive targeted therapy because no apparent actionable alterations are detected, but additionally, the difficulty of obtaining sufficient material for molecular profiling remains a challenge [16]. However, for around 60% of the BTC patients, chemotherapy remains the only therapeutic option. Therefore, second-line systemic therapy in aBTCs represents an area of significant and growing unmet need in oncology. In this retrospective, multicenter analysis based on the experience of three Italian oncologic centers, our aim was to evaluate outcomes and prognostic parameters in advanced ICC patients treated with second-line chemotherapy. 

## 2. Materials and Methods

### 2.1. Patients, Data Collection, and Follow-Up 

This study protocol was reviewed and approved by the Ethics Committee “Area Vasta Emilia Nord” (protocol 78/2017/O/OSSN), which was in accordance with the Declaration of Helsinki and Good Clinical Practice. It was conducted in three Italian Hospitals (Parma, Modena and Bologna). A total of 255 patient files were retrieved using electronic medical records. All consecutive patients with histologically proven metastatic intrahepatic cholangiocarcinomas treated with first-line and second-line palliative chemotherapy between January 2012 and December 2018 were eligible for this study. The biliary tract cancers consisting of intrahepatic cholangiocarcinomas, extrahepatic cholangiocarcinoma, gallbladder, or ampullary cancers were excluded from this study. The patients were treated with first-line chemotherapy until disease progression or unacceptable toxicity. The main regimen for the first-line treatment was the combination of gemcitabine and cisplatin (gemcitabine at 1000 mg/m^2^ and cisplatin at 25 mg/m^2^, each on days 1 and 8 of a 21-day regimen) or gemcitabine and oxaliplatin (gemcitabine at 1000 mg/m^2^ and oxaliplatin at 100 or 85 mg/m^2^, every 2 weeks). In the case of unacceptable toxicities after platinum-based chemotherapy doublet, monotherapy without platinum derived was continued until disease progression when second-line chemotherapy was introduced. The main regimens used for second-line chemotherapy were 5-fluoropyrimidine-based doublets such as XELOX/FOLFOX, XELIRI/FOLFIRI, or capecitabine/De Gramont regimen. The regimens were as follows: FOLFOX (5-FU bolus at 400 mg/m^2^ on day 1, and 5-FU infusion at 2400 mg/m^2^ over the course of 46 h every 2 weeks) plus oxaliplatin at 100 mg/m^2^ on day 1 every 2 weeks), XELOX (capecitabine at 2000 mg/m^2^/day on days 1–14 and oxaliplatin at 130 mg/m^2^ on day 1 every 3 weeks), FOLFIRI (5-FU bolus at 400 mg/m^2^ on day 1, and 5-FU infusion at 2400 mg/m^2^ over the course of 46 h every 2 weeks) plus irinotecan at 180 mg/m^2^ on day 1), XELIRI (capecitabine at 2000 mg/m^2^/day on days 1–14 and irinotecan at 200 mg/m^2^ on day 1 every 3 weeks), capecitabine (2000 mg/m^2^/day on days 1–14 every 3 weeks) and De Gramont regimen (5-FU bolus at 400 mg/m^2^ on day 1, and 5-FU infusion at 2400 mg/m^2^ over the course of 46 h every 2 weeks). All patients were evaluated with computed tomography (CT) or magnetic resonance scan every 3 months during first-line and second-line treatment. According to the RECIST evaluation (https://recist.eortc.org/ accesses on 15 July 2023), tumor response was classified as a progressive disease, stable disease, or complete or partial remission, corresponding to the evaluation criteria in solid tumors (RECIST, version 1.1). Baseline clinical and pathological parameters were recorded prior to palliative chemotherapy start. PFS is defined as the time period from beginning first-line chemotherapy (PFS1) or second-line chemotherapy (PFS2) until disease progression or death. We documented patients’ outcomes (PFS1, PFS2, date of death, or last follow-up), as well as Best Response to first-line chemotherapy and the type of second-line treatment.

### 2.2. Statistical Analysis

**Figure 1:**
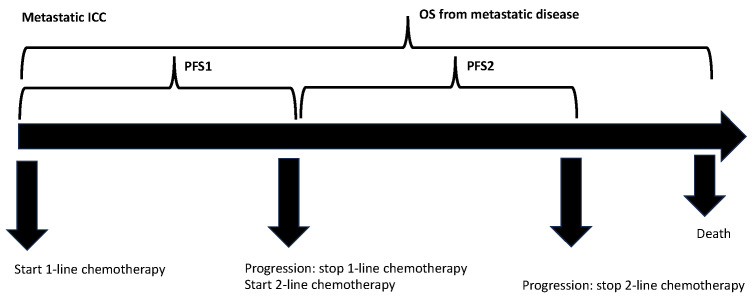
Schematic view of PFS1, PFS2, and OS: PFS was defined as the time period from beginning first-line chemotherapy (PFS1) or second-line chemotherapy (PFS2) until disease progression. OS for metastatic disease was defined as the time period from the application of first-line palliative chemotherapy until death.

Clinical and pathologic, as well as long-term survival data, were collected and reviewed. OS from metastatic disease was calculated from the date of metastatic ICC diagnosis to the date of death. PFS after first-line and second-line chemotherapy (PFS1 and PFS2) were defined as the time of first-line or second-line therapy initiation to disease progression or death. Best Response to first-line therapy was divided into progressive and non-progressive disease (including stable disease and partial or complete remission). OS, PFS1, and PFS2 curves were compared by log-rank test and transcribed into Kaplan-Meier diagrams. OS for metastatic disease is defined as the time period from the application of first-line palliative chemotherapy until death. Schematic view of PFS1 and PFS2 are shown in Figure 1. Univariate and multivariate analyses were performed using Cox regression models. Variables that showed significant *p*-values in univariate analysis (age, sex, ECOG PS, surgery, PFS1, Best Response to first-line chemotherapy, PFS2, and the type of second-line chemotherapy (gemcitabine-based versus fluoropyrimidine-based) were included in multivariate Cox regression analysis. IBM SPSS Statistics v 25.0 (IBM) was used to perform all computational analyses. The *p*-value was bilaterally tested, and values less than 0.05 were regarded as statistically significant. 

## 3. Results

### 3.1. Population Characteristics 

Out of 255 consecutive metastatic ICC patients included in our study, 143 (55.4%) were males and 115 (44.6%) were females. The median age at diagnosis was 66.5 years (range 34–86 years). Fourty-four percent (113/255) of metastatic ICC patients underwent second-line chemotherapy. The median of PFS2 in patients treated with second-line chemotherapy was 3 months (range 1–30 months). In the whole group, the median OS from metastatic disease occurrence was 11 months (range 1–1471 months).

The basic clinical and pathological characteristics of the 255 ICC patients are detailed in Table 1. As demonstrated in Table 1, statistically significant differences were seen among the two groups who received or did not receive second-line chemotherapy for age, ECOG PS, PFS1, and Best Response to first-line chemotherapy. In particular, younger ICC patients with better ECOG PS status who responded to first-line chemotherapy were those who were treated with second-line treatment.

### 3.2. Overall Survival for Metastatic Disease Depends on PFS1 and PFS2

**Figure 2:**
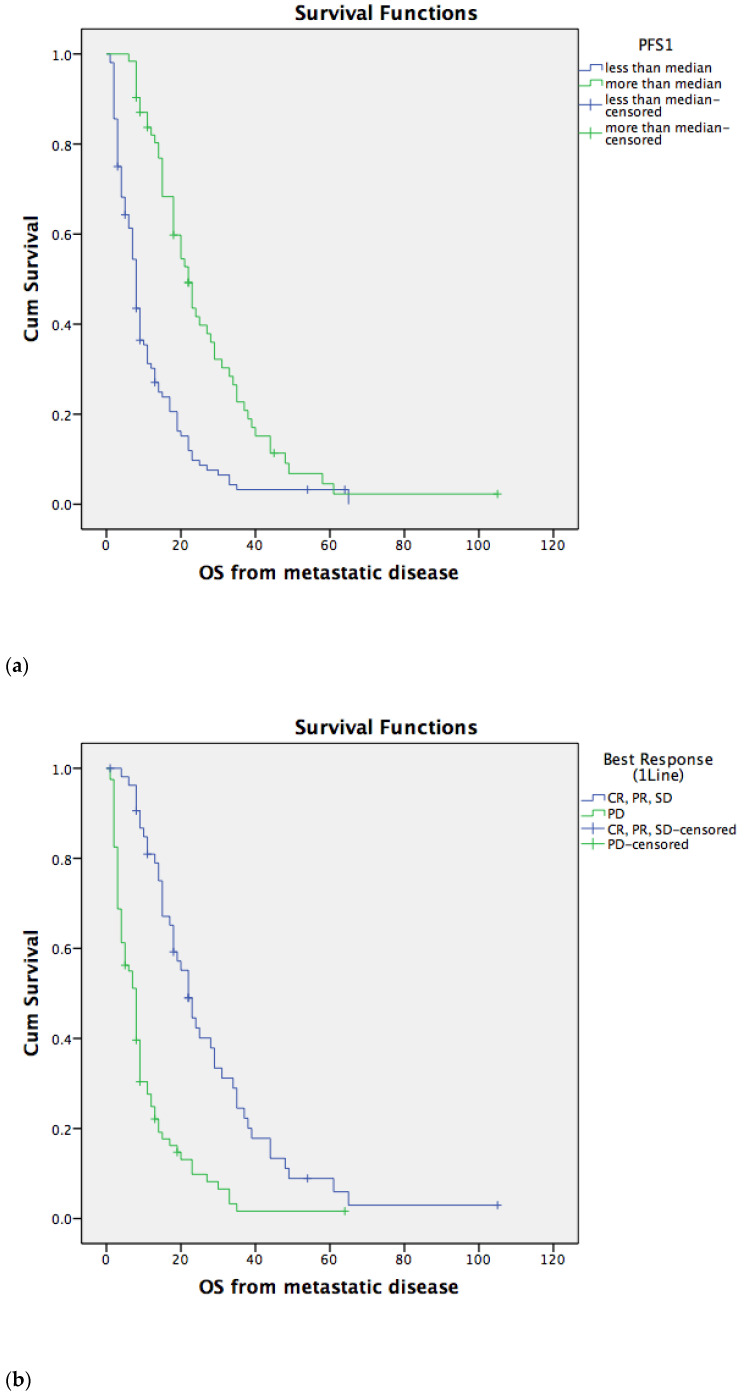

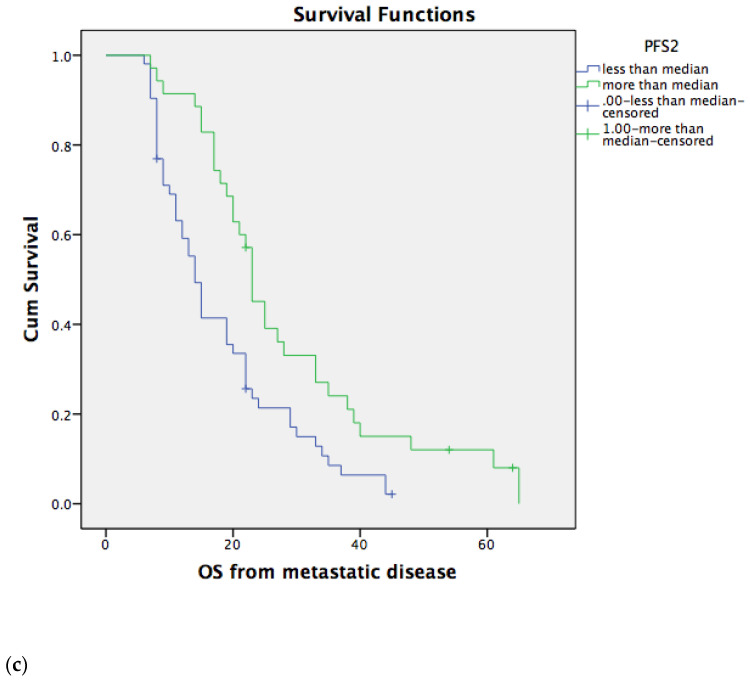
(**a**) Median OS of ICC patients depends on PFS1. The ICC patients with longer responses to first-line chemotherapy had a longer OS, *p* < 0.001. (**b**) Median OS depended on disease control after first-line chemotherapy. The patients with disease control after first-line chemotherapy had a longer OS, *p* < 0.001. (**c**) Median OS depended on PFS2, the patients with longer response to second-line chemotherapy had a longer OS, *p* = 0.002.

We divided patients into two groups according to PFS1: with PFS1 less or more than the median. ICC patients with a shorter median PFS1 have a worse median OS (8 months, range 7–9 months) compared to ICC patients with a longer than median PFS1 (median OS was 22 months, ranging from 18–26 months), with a statistically significant difference (*p* < 0.001), as demonstrated in Figure 2b. Moreover, ICC patients who had progressive disease after first-line chemotherapy also had a worse median OS (8 months, ranging from 6–10 months) compared to ICC patients with no progressive disease as the best response to first-line chemotherapy (a median OS of 22 months, ranging from 18–26 months), with a statistically significant difference (*p* < 0.001), as demonstrated in Figure 2b. The median PFS2 was 3 months (range 1–30 months). Again, we divided patients into two groups according to PFS2: with PFS2 less or more than the median. ICC patients who underwent second-line chemotherapy and had a shorter median PFS2 also had worse median OS (14 months, ranging from 12–16 months) compared to ICC patients with a longer than median PFS2 (the median OS was 23 months, ranging from 20–26 months), with a statistically significant difference (*p* = 0.002) as demonstrated in Figure 2c. 

### 3.3. Overall Survival for Metastatic Disease Does Not Depend on the Type of Second-Line Chemotherapy

We evaluated the type of second-line chemotherapy treatment (fluoropyrimidine-based or gemcitabine-based). The majority of the patients (66%) were treated with fluoropyrimidine-based chemotherapy, and the rest were treated with gemcitabine-based chemotherapy. Interestingly, no statistically significant differences were seen between the two groups according to the type of second-line chemotherapy (*p* = 0.93).

### 3.4. Univariate and Multivariate Prognostic Analyses

To outline the clinically relevant factors associated with predicting the impact on OS, we used univariate and multivariate COX regression models (Table 2 and Table 3). Univariate analysis revealed that a shorter than median PFS1 and PFS2 were associated with a significantly higher Hazard Ratio (HR) for death (HR = 2.5; 95%CI = 1.8–3.6; *p* < 0.001, HR = 1.9; 95%CI = 1.2–3.1; *p* = 0.004) (Table 2). On the contrary, a better ECOG PS and disease control after the first-line chemotherapy were associated with a significantly lower HR for death (HR = 0.17; 95%CI = 0.10–0.26; *p* < 0.001, HR = 0.32; 95%CI = 0.21–0.47, *p* < 0.001). We added all of these variables to the multivariate analysis. For the Cox analysis, the following reference groups were selected: the median age less than the median, male sex, ECOG PS 0–1 group, no surgery group, median PFS1 less than the median, Best Response to first-line chemotherapy with disease control, gemcitabine-based second-line chemotherapy, and median PFS2 less than the median. The Cox proportional hazards model analysis showed that no surgery and a median PFS2 less than the median were associated with a significantly higher HR for death (HR = 3.8; 95%CI = 1.58–9.17; *p* = 0.003 and HR = 3.4; 95%CI = 1.67–7.26; *p* = 0.001, respectively). Younger age was also associated with a higher HR for death, with a tendency for a statistically significant difference (HR = 1.7, CI = 0.96–3.31; *p* = 0.06). In contrast, ECOG PS 0–1 group and Best Response to first-line chemotherapy with no progressive disease were associated with a significantly lower HR for death (HR = 0.05; 95%CI = 0.009–0.29; *p* = 0.001 and HR = 0.2; 95%CI = 0.07–0.56; *p* = 0.002, respectively), see Table 3.

## 4. Discussion

In our series, 44% of ICC patients were treated with second-line chemotherapy. In particular, younger ICC patients with better ECOG PS status, with disease control after first-line chemotherapy, were those who underwent second-line treatments. Further, the ICC patients with better ECOG PS with prior surgical resection of the primary tumor who responded to first-line chemotherapy and had better PFS2 were associated with a better outcome in multivariate analysis.

Even if the disease were rare, the incidence of ICC has demonstrated an increasing tendency in recent decades. According to some authors, since the early 1990s, there has been a 14% increase in incidence per year [19,20]. The prognosis of ICC patients is more favorable compared with other aBTC as demonstrated in post hoc analysis of the ABC-01, -02, and -03 clinical trials [21]. For these reasons, it is important to search for new therapeutic possibilities in this subgroup of aBTC. Surgical resection is the only potentially curative option for BTC. However, due to their aggressiveness, the majority of patients (>65%) are diagnosed with non-resectable diseases. For these patients, systemic treatment with palliative intent remains the current treatment of choice. Nowadays, the treatment options for advanced ICC do not differ from other aBTC; the combination of gemcitabine and cisplatin was the first-line standard of care from 2010 based on the survival benefit shown with this regimen over gemcitabine alone in the ABC-02 clinical trial [6]. In this study, 410 patients affected by aBTC (ICC, ECC, gallbladder, and ampullary cancers) were enrolled. A total of 204 patients received doublet treatment (cisplatin plus gemcitabine) and 206 patients were treated with gemcitabine monotherapy. The median OS was 11.7 months among the 204 patients in the cisplatin–gemcitabine group and 8.1 months among the 206 patients in the gemcitabine group (hazard ratio, 0.64; 95% confidence interval, 0.52 to 0.80; *p* < 0.001). The median PFS was 8.0 months in the cisplatin–gemcitabine group and 5.0 months in the gemcitabine-only group (*p* < 0.001). In addition, the response rate among patients treated with doubled chemotherapy was higher compared to the patients’ group treated with gemcitabine in monotherapy (81.4% vs. 71.8%, *p* = 0.049) [6]. Cholangiocarcinomas exhibit expression of the immune checkpoint molecules, programmed cell death ligand 1 (PD-L1), and cytokine T-lymphocyte–associated protein 4 (CTLA-4), in the tumor microenvironment. In fact, the clinical activity of immune checkpoint inhibitors in aBTC, including durvalumab, a PD-L1 inhibitor, has been demonstrated in a recent TOPAZ-1 trial. The TOPAZ-1 trial compared the combination of gemcitabine and cisplatin, combined with durvalumab/placebo. The addition of durvalumab to the old chemotherapy backbone became a new standard of care for the first-line palliative setting [7]. In the TOPAZ-1 trial, a total of 685 patients affected by both unresectable locally advanced or metastatic adenocarcinoma of the biliary tract (including ICC, ECC, and gallbladder carcinoma) were enrolled. The percentage of ICC, ECC, and gallbladder cancer patients were well balanced between the two arms. In the TOPAZ-1 trial, the Kaplan–Meier curve for OS Kaplan was separated at approximately 6 months of therapy, after which there was a clear and sustained separation of the survival curves in favor of the durvalumab group. Unfortunately, nowadays, we do not have any biomarkers that would predict the efficacy of or resistance to durvalumab. The median OS was 12.8 months in the durvalumab group and 11.5 months in the placebo treatment group. Moreover, the median PFS was 7.2 months with durvalumab and 5.7 months with placebo [7]. However, disease progression inevitably occurs during front-line chemotherapy, resulting in an OS hardly exceeding one year [22,23,24]. 

Until 2019, there was no recommended regimen in the second-line setting. It has been estimated that approximately 15–25% of aBTC receive second-line therapy due to rapidly worsening performance status after first-line treatment [11,14,25]. In our study, evaluating only ICC patients, 44% of patients underwent second-line chemotherapy. This is probably due to a more favorable prognosis of ICC, characterized by different natural history compared to another aBTC. In the last few years, the phase III ABC-06 trial in the non-Asian population and the phase II NIFTY trial in the Asian population demonstrated a statistically significant improvement in PFS and OS after the standard first-line treatment [14,26]. The ABC-06 phase 3 clinical trial enrolled both locally advanced or metastatic biliary tract cancer (including ICC, ECC, gallbladder, or ampullary carcinoma) with documented radiological disease progression to first-line standard doublet chemotherapy (cisplatin and gemcitabine), still in good general clinical conditions (ECOG PS 0–1). The patients were randomized to active anticancer chemotherapy treatment with FOLFOX and Best Supportive Care (BSC) versus BSC only. The OS was significantly longer in the FOLFOX with BSC group than in the BSC alone group, with a median OS of 6.2 months in the FOLFOX with BSC group versus 5.3 months in the BSC alone group (adjusted hazard ratio 0.69 [95% CI 0.50–0.97]; *p* = 0.031). The results of the ABC-06 clinical trial demonstrated that FOLFOX chemotherapy can improve the OS in patients with good ECOG PS with aBTC who have been previously treated with cisplatin and gemcitabine. Therefore, in Western countries, FOLFOX became a standard of care in second-line settings [14]. The improvement in OS is though very modest. In particular, the addition of FOLFOX in the ABC-06 trial showed a median PFS of 4 months and improved OS of less than 1 month (HR = 0.69, *p* = 0.031) [14]. The NIFTY trial was a randomized, phase 2b clinical trial conducted in Asian countries. A total of 178 patients with aBTC, whose disease progressed while receiving first-line gemcitabine plus cisplatin chemotherapy, were eligible for this study. Patients were randomized to receive the De Gramont regimen with or without liposomal irinotecan and were treated until disease progression or unacceptable toxic effects. The updated analysis reported a median PFS of 4.2 months for a doublet chemotherapy regimen with liposomal irinotecan and 1.7 months for the De Gramont regimen alone group, respectively (hazard ratio, 0.61; 95% CI, 0.44–0.86; *p* = 0.004) [14]. Both the ABC-06 trial in the non-Asian population and the NIFTY trial in the Asian population showed that there is only a modest PFS of 4 months with active doublet anti-cancer treatment in the second-line setting of aBTC patients.

The comparison with fluoropyrimidine monotherapy was not available in the ABC-06 trial. Several authors suggest that fluoropyrimidine monotherapy is as active as fluoropyrimidine doublets in aBTC in second-line settings [27,28]. The French-Italian study aimed to assess the OS of aBTC patients treated in second-line with a fluoropyrimidines monotherapy compared to a doublet with irinotecan or platinum. The authors performed a retrospective analysis of two large multicenter cohorts: a French cohort and an Italian cohort. In the French cohort (351 patients), no significant OS difference was observed between the fluoropyrimidines monotherapy and doublet groups (median OS 5.6 vs. 6.8 months, *p* = 0.65). Similar findings were observed in the Italian cohort (174 patients). The authors concluded that fluoropyrimidine monotherapy is as active as fluoropyrimidine doublets in aBTC in second-line, regardless of the patient performance status and country, and could be a therapeutic option in this setting [11]. A similar retrospective study was conducted by Kim et al. [25]. The authors examined patients affected by aBTC who received first-line gemcitabine and cisplatin chemotherapy. Among 748 patients treated with first-line gemcitabine and cisplatin, 321 (43%) subsequently received fluoropyrimidine-based second-line systemic chemotherapy which is in accordance with our study with 44% of ICC patients treated with second-line chemotherapy. Fluoropyrimidine monotherapy and fluoropyrimidine–platinum doublet combination were used in 255 and 66 patients, respectively. The median PFS and OS were 1.9 months and 6.5 months, respectively. The overall response rate was significantly higher in patients who received fluoropyrimidine–platinum doublet combination compared with those who received fluoropyrimidine alone (8 vs. 1%, *p* = 0.009), although the PFS (*p* = 0.43) and OS (*p* = 0.88) did not significantly differ between these groups. Kim et al. concluded that fluoropyrimidine–platinum combination therapy was not associated with improved survival outcomes, as compared with fluoropyrimidine monotherapy [25].

Regarding the reported efficacy of second-line chemotherapy, the median PFS in ICC patients who received second-line treatment in our study was 3 months. Similar results were described by Lamarca et al. who evaluated 761 aBTC patients [12]. This systematic review reported a mean PFS of 3.2 months [12]. Ying et al. evaluated the efficacy of second-line therapy in 1391 aBTC and reported a median PFS of 2.6 months in patients who received second-line treatment [29] similar to the median PFS of our study. Moreover, the authors evaluated the efficacy of different regimens (in particular fluoropyrimidine-based, gemcitabine-based, or taxanes-based chemotherapy doublets or monotherapy). The combination therapy was not superior to single-agent therapy in terms of overall response rate, disease-control rate, and 1-year OS, and fluoropyrimidine-based chemotherapy seems to be inferior to other second-line regimens [29]. Moreover, Fornaro et al. [30] described response rate with second-line chemotherapy was low (3.4%), with a median PFS and OS of 3.0 months and 6.6 months, respectively. In order to identify an optimal chemotherapeutic approach in second-line, Fornaro et al. compared the outcome of aBTC patients receiving monotherapy with that of patients treated with a combination regimen. No imbalances in the main patient characteristics between the two groups were identified. A slight increase in disease-control rate (32% vs. 21%, *p* = 0.140) and PFS (median: 3.1 vs. 2.9 months; *p* = 0.072) was observed, but these differences did not reach statistical significance. Of note, contrary to previous studies, they reported prolonged OS with combination chemotherapy compared to single-agent (7.1 vs. 5.0 months; *p* = 0.006).

Patients with aBTC treated with second-line chemotherapy display prognostic heterogeneity [12,27,28,30]. Several authors aimed to analyze second-line treatments in aBTC. Neuzillet et al. analyzed nearly 800 patients with aBTC from four independent cohorts [31]. The authors showed that not all patients benefit from second-line chemotherapy, and that altered ECOG PS is the strongest risk factor associated with shorter OS in second-line setting [31]. Moreover, they also identified an absence of primary tumor resection, first-line chemotherapy discontinuation for progression, high CA19-9 serum level, and the presence of peritoneal carcinomatosis as other independent prognostic biomarkers for OS in this setting [31]. The presence of bone metastases was associated with a worse outcome for ICC patients in multivariate analysis [3]. Fornaro et al. [30] investigated 300 consecutive aBTC patients and identified four parameters, independently associated with longer OS in patients undergoing second-line chemotherapy: ECOG PS, pretreatment CA19-9 serum level, PFS after first-line chemotherapy more than 6 months, and previous surgery on the primary tumor. This is in accordance with our study where the ICC patients with better ECOG PS, with prior primary tumor resection, and better efficacy of first-line chemotherapy were associated with a better outcome in multivariate analysis. Further, Brieau et al. [32] performed a multicenter study to describe the second-line regimens used, the response rates, and the outcomes of patients treated with various regimens. Out of 603 patients who received first-line chemotherapy for aBTC, 196 received second-line chemotherapy with the median PFS and OS were 3.2 and 6.7 months, respectively [32]. Interestingly, and in accordance with our study, there was no significant difference in PFS or OS between various regimens. However, it should be underlined that in our study, the second-line treatment was based on the physician’s choice and therefore represents a bias. In a multivariate analysis, ECOG PS, disease control with first-line chemotherapy, and CA 19-9 serum levels were significantly associated with longer PFS and OS [32].

Our study is limited by its retrospective nature for data collection and the high rate of missing data for biological data, though the evaluated ICC population is more homogeneous compared to other studies which included all aBTC. Moreover, another limitation of our study was that routine molecular profiling was not available for all patients participating in our study because it was not the standard of care at the time of the study’s conduct; thus, the impact on patients with specific molecular aberrations is not known [18]. Nowadays, the ESMO Scale for Clinical Actionability of Molecular Targets (ESCAT) ranks a match between drug and genomic alterations, according to their actionability [18]. Nowadays, in aBTC, tumor multigene NGS should be used to detect level I actionable alterations in cholangiocarcinoma during or after first-line chemotherapy progression. ESCAT level I means that the match of an alteration and a drug has been validated in clinical trials, and should drive treatment decisions in daily practice [18]. Advanced BTCs have four level I genomic alterations: (1) IDH1 mutations (20%), (2) FGFR2 fusions (15%), (3) NTRAK fusions (2%), and (4) MSI-H (2%) [13,15,18]. These alterations are targetable and the use of personalized second-line therapy is recommended in these cases instead of second-line chemotherapy. Even if targeted agents in a second-line setting are only indicated for a small fraction of BTC patients whose tumors harbor specific genomic alterations, their use demonstrated a significant outcome benefit. The randomized phase III ClarIDHy trial, which enrolled pre-treated BTC patients and IDH 1/2 mutations, found a statistically significant improvement in PFS for patients receiving the IDH1 inhibitor ivosidenib compared with the placebo group (median PFS 2.7 vs. 1.4 months; HR, 0.37) [33]. Regarding FGFR gene rearrangements, both pemigatinib and infigratinib showed impressive median OS of 17.5 and 12.2 months, respectively [15,34]. Two NTRAK inhibitors, namely entrectinib and larotrectinib, have a tumor-agnostic indication for patients with unresectable, locally advanced, or metastatic solid tumors that have confirmed NTRK gene fusions, and no other satisfactory treatment options [35,36]. Moreover, BTC patients who harbor other actionable genomic alterations such as RET, BRAF, HER2, and MSI, should be considered for targeted therapy through clinical trials or other means of access [37,38]. Unfortunately, only a minority of BTC patients harbor specific genomic alterations and for the majority of aBTC (around 60%), chemotherapy remains the only therapeutic option; therefore the efficacy of second-line chemotherapy remains a clinically relevant topic.

In conclusion, in our study, we evaluated only ICC patients, a more homogeneous group compared to most studies that evaluated all aBTC (including ECC, gallbladder, and ampullary cancers). In our series, approximately 44% of ICC patients underwent second-line palliative treatment. Not all patients seem to benefit from second-line chemotherapy and it has to be evaluated on an individual basis because the prognosis remains poor. No particular regimen seems to be superior to others in our series, and this underlines the need for new treatments. To improve therapeutic decisions, performance status and disease control with first-line chemotherapy should lead the decision on the usefulness of second-line treatments in advanced ICC patients, though randomized clinical trials are needed to confirm this.

## Figures and Tables

**Table 1:** Basic clinical and pathological characteristics of the ICC patients.

Variable	NO Second-Line CT (pts)	YES Second-Line CT (pts)	*p*
Number of patients	142	113	
Median Age (66.5 years)			
less than median	62/142 (44%)	68/113 (60%)	0.006
more than median	80/142 (56%)	45/113 (40%)	
Sex			
male	82/142 (58%)	59/113 (52%)	0.215
female	60/142 (42%)	54/113 (48%)	
ECOG PS			
0–1	98/142 (69%)	108/113 (96%)	<0.001
>2	35/142 (25%)	4/113 (3%)	
unknown	9/142 (6%)	1/113 (1%)	
Surgery for primary tumor			
yes	42/142 (30%)	39/113 (35%)	0.255
no	100/142 (70%)	74/113 (65%)	
PFS1 (4 months)			
less than median	65/142 (46%)	58/113 (51%)	0.002
more than median	25/142 (18%)	55/113 (49%)	
unknown	52/142 (36%)		
Best Response to 1-line CT			
SD, PR, CR	14/142 (10%)	42/113 (37%)	<0.001
PD	53/142 (37%)	29/113 (26%)	
unknown	75/142 (53%)	42/113 (37%)	
2-line CT G vs. FU			
gemcitabine-based		34/113 (30%)	
FU-based		66/113 (58%)	
unknown		13/113 (12%)	
PFS2 (3 months)			
less than median		62/113 (55%)	
more than median		42/113 (37%)	
unknown		9/113 (8%)	

CT = chemotherapy; pts = patients; PFS1 = progression-free survival for first-line chemotherapy; PFS2 = progression-free survival for second-line chemotherapy; G = gemcitabine; FU = fluoropyrimidine.

**Table 2:** Univariate Cox analysis of metastatic overall survival.

Variable	HR	95.0% CI for HR	*p*
Median age (reference: median age less than the median)	1.165	0.862–1.575	0.322
Sex (reference: male sex)	1.241	0.918–1.676	0.160
ECOG PS (reference: ECOG PS 0–1 group)	0.170	0.108–0.266	<0.001
Surgery for primary tumor (reference: no surgery)	1.099	0.801–1.508	0.557
PFS1 (reference: PFS1 less than the median)	2.570	1.824–3.620	<0.001
Best Response to 1-line CT (reference: disease control)	0.324	0.219–0.479	<0.001
2-line CT G vs. FU (reference: 2-line CT G)	1.006	0.603–1.677	0.983
PFS2 (reference: PFS2 less than the median)	1.987	1.249–3.161	0.004

CT = chemotherapy; pts = patients; PFS1 = progression-free survival for first-line chemotherapy; PFS2 = progression-free survival for second-line chemotherapy; G = gemcitabine; FU = fluoropyrimidine; HR = hazard ratio.

**Table 3:** Multivariate Cox analysis of metastatic overall survival.

Variable	HR	95.0% CI for HR	*p*
Median age (reference: median age less than the median)	1.785	0.962–3.313	0.06
Sex (reference: male sex)	1.651	0.883–3.089	0.11
ECOG PS (reference: ECOG PS 0–1 group)	0.051	0.009–0.292	0.001
Surgery for primary tumor (reference: no surgery)	3.812	1.584–9.174	0.003
PFS1 (reference: PFS1 less than the median)	0.571	0.228–1.430	0.23
Best Response to 1-line CT (reference: disease control)	0.203	0.073–0.560	0.002
2-line CT G vs. FU (reference: 2-line CT G)	1.008	0.463–2.190	0.98
PFS2 (reference: PFS2 less than the median)	3.494	1.679–7.269	0.001

CT = chemotherapy; pts = patients; PFS1 = progression-free survival for first-line chemotherapy; PFS2 = progression-free survival for second-line chemotherapy; G = gemcitabine; FU = fluoropyrimidine; HR = hazard ratio.

## Data Availability

The data presented in this study are available on request from the corresponding author. The data are not publicly available due to privacy.

## References

[1] Vogel A., Bridgewater J., Edeline J., Kelley R.K., Klümpen H.J., Malka D., Primrose J.N., Rimassa L., Stenzinger A., Valle J.W. (2023). Biliary Tract Cancer: ESMO Clinical Practice Guideline for Diagnosis, Treatment and Follow-Up. Ann. Oncol..

[2] Valle J.W., Kelley R.K., Nervi B., Oh D.-Y., Zhu A.X. (2021). Biliary Tract Cancer. Lancet.

[3] Garajová I., Gelsomino F., Salati M., Leonardi F., De Lorenzo S., Granito A., Tovoli F. (2023). Bone Metastases from Intrahepatic Cholangiocarcinoma Confer Worse Prognosis. Curr. Oncol..

[4] Tovoli F., Garajová I., Gelsomino F., Iavarone M., Federico P., Salati M., Corianò M., Caputo F., De Lorenzo S., Granito A. (2022). Pattern of Progression of Intrahepatic Cholangiocarcinoma: Implications for Second-Line Clinical Trials. Liver Int..

[5] De Lorenzo S., Garajova I., Stefanini B., Tovoli F. (2021). Targeted Therapies for Gallbladder Cancer: An Overview of Agents in Preclinical and Clinical Development. Expert Opin. Investig. Drugs.

[6] Valle J., Wasan H., Palmer D.H., Cunningham D., Anthoney A., Maraveyas A., Madhusudan S., Iveson T., Hughes S., Pereira S.P. (2010). Cisplatin plus Gemcitabine versus Gemcitabine for Biliary Tract Cancer. N Engl. J. Med..

[7] Oh D.-Y., He A.R., Qin S., Chen L.-T., Okusaka T., Vogel A., Kim J.W., Suksombooncharoen T., Lee M.A., Kitano M. (2022). A Phase 3 Randomized, Double-Blind, Placebo-Controlled Study of Durvalumab in Combination with Gemcitabine plus Cisplatin (GemCis) in Patients (Pts) with Advanced Biliary Tract Cancer (BTC): TOPAZ-1. J. Clin. Oncol..

[8] Oh D.-Y., He A.R., Qin S., Chen L.-T., Okusaka T., Vogel A., Kim J.W., Suksombooncharoen T., Lee M.A., Kitano M. (2022). 56P Updated Overall Survival (OS) from the Phase III TOPAZ-1 Study of Durvalumab (D) or Placebo (PBO) plus Gemcitabine and Cisplatin (+ GC) in Patients (Pts) with Advanced Biliary Tract Cancer (BTC). Ann. Oncol..

[9] Oh D.-Y., Ruth He A., Qin S., Chen L.-T., Okusaka T., Vogel A., Kim J.W., Suksombooncharoen T., Ah Lee M., Kitano M. (2022). Durvalumab plus Gemcitabine and Cisplatin in Advanced Biliary Tract Cancer. NEJM Evid..

[10] Kelley R.K., Ueno M., Yoo C., Finn R.S., Furuse J., Ren Z., Yau T., Klümpen H.J., Chan S.L., Ozaka M. (2023). Pembrolizumab in Combination with Gemcitabine and Cisplatin Compared with Gemcitabine and Cisplatin Alone for Patients with Advanced Biliary Tract Cancer (KEYNOTE-966): A Randomised, Double-Blind, Placebo-Controlled, Phase 3 Trial. Lancet.

[11] Walter T., Horgan A.M., McNamara M., McKeever L., Min T., Hedley D., Serra S., Krzyzanowska M.K., Chen E., Mackay H. (2013). Feasibility and Benefits of Second-Line Chemotherapy in Advanced Biliary Tract Cancer: A Large Retrospective Study. Eur. J. Cancer.

[12] Lamarca A., Hubner R.A., David Ryder W., Valle J.W. (2014). Second-Line Chemotherapy in Advanced Biliary Cancer: A Systematic Review. Ann. Oncol..

[13] Lowery M.A., Goff L.W., Keenan B.P., Jordan E., Wang R., Bocobo A.G., Chou J.F., O’Reilly E.M., Harding J.J., Kemeny N. (2019). Second-line Chemotherapy in Advanced Biliary Cancers: A Retrospective, Multicenter Analysis of Outcomes. Cancer.

[14] Lamarca A., Palmer D.H., Wasan H.S., Ross P.J., Ma Y.T., Arora A., Falk S., Gillmore R., Wadsley J., Patel K. (2021). Second-Line FOLFOX Chemotherapy versus Active Symptom Control for Advanced Biliary Tract Cancer (ABC-06): A Phase 3, Open-Label, Randomised, Controlled Trial. Lancet Oncol..

[15] Abou-Alfa G.K., Sahai V., Hollebecque A., Vaccaro G., Melisi D., Al-Rajabi R., Paulson A.S., Borad M.J., Gallinson D., Murphy A.G. (2020). Pemigatinib for Previously Treated, Locally Advanced or Metastatic Cholangiocarcinoma: A Multicentre, Open-Label, Phase 2 Study. Lancet Oncol..

[16] Rushbrook S.M., Kendall T.J., Zen Y., Albazaz R., Manoharan P., Pereira S.P., Sturgess R., Davidson B.R., Malik H.Z., Manas D. (2023). British Society of Gastroenterology guidelines for the diagnosis and management of cholangiocarcinoma. Gut.

[17] Jusakul A., Cutcutache I., Yong C.H., Lim J.Q., Huang M.N., Padmanabhan N., Nellore V., Kongpetch S., Ng A.W.T., Ng L.M. (2017). Whole-Genome and Epigenomic Landscapes of Etiologically Distinct Subtypes of Cholangiocarcinoma. Cancer Discov..

[18] Mosele F., Remon J., Mateo J., Westphalen C.B., Barlesi F., Lolkema M.P., Normanno N., Scarpa A., Robson M., Meric-Bernstam F. (2020). Recommendations for the Use of Next-Generation Sequencing (NGS) for Patients with Metastatic Cancers: A Report from the ESMO Precision Medicine Working Group. Ann. Oncol..

[19] Acher A.W., Paro A., Elfadaly A., Tsilimigras D., Pawlik T.M. (2021). Intrahepatic Cholangiocarcinoma: A Summative Review of Biomarkers and Targeted Therapies. Cancers.

[20] Endo I., Gonen M., Yopp A.C., Dalal K.M., Zhou Q., Klimstra D., D’Angelica M., Dematteo R.P., Fong Y., Schwartz L. (2008). Intrahepatic Cholangiocarcinoma: Rising Frequency, Improved Survival, and Determinants of Outcome after Resection. Ann. Surg..

[21] Lamarca A., Ross P., Wasan H.S., Hubner R.A., McNamara M.G., Lopes A., Manoharan P., Palmer D., Bridgewater J., Valle J.W. (2020). Advanced Intrahepatic Cholangiocarcinoma: Post Hoc Analysis of the ABC-01, -02, and -03 Clinical Trials. J. Natl. Cancer Inst..

[22] Adeva J., Sangro B., Salati M., Edeline J., La Casta A., Bittoni A., Berardi R., Bruix J., Valle J.W. (2019). Medical Treatment for Cholangiocarcinoma. Liver Int..

[23] Rizzo A., Salati M., Frega G., Merz V., Caputo F., Ricci A.D., Palloni A., Messina C., Spallanzani A., Saccoccio G. (2021). Second-Line Chemotherapy (2L) in Elderly Patients with Advanced Biliary Tract Cancer (ABC): A Multicenter Real-World Study. J. Clin. Oncol..

[24] Rizzo A., Salati M., Frega G., Merz V., Caputo F., Di Federico A., Palloni A., Carloni R., Ricci A.D., Gadaleta-Caldarola G. (2022). Second-Line Chemotherapy in Elderly Patients with Advanced Biliary Tract Cancer: A Multicenter Real-World Study. Medicina.

[25] Bridgewater J., Palmer D., Cunningham D., Iveson T., Gillmore R., Waters J., Harrison M., Wasan H., Corrie P., Valle J. (2013). Outcome of Second-Line Chemotherapy for Biliary Tract Cancer. Eur. J. Cancer.

[26] Yoo C., Kim K.p., Jeong J.H., Kim I., Kang M.J., Cheon J., Kang B.W., Ryu H., Lee J.S., Kim K.W. (2021). Liposomal Irinotecan plus Fluorouracil and Leucovorin versus Fluorouracil and Leucovorin for Metastatic Biliary Tract Cancer after Progression on Gemcitabine plus Cisplatin (NIFTY): A Multicentre, Open-Label, Randomised, Phase 2b Study. Lancet Oncol..

[27] Neuzillet C., Casadei-Gardini A., Brieau B., Vivaldi C., Brandi G., Tougeron D., Filippi R., Vienot A., Silvestris N., Pointet A.L. (2020). Fluropyrimidine Single Agent or Doublet Chemotherapy as Second Line Treatment in Advanced Biliary Tract Cancer. Int. J. Cancer.

[28] Kim B.J., Yoo C., Kim K.P., Hyung J., Park S.J., Ryoo B.Y., Chang H.M. (2017). Efficacy of Fluoropyrimidine-Based Chemotherapy in Patients with Advanced Biliary Tract Cancer after Failure of Gemcitabine plus Cisplatin: Retrospective Analysis of 321 Patients. Br. J. Cancer.

[29] Ying J., Chen J. (2019). Combination versus Mono-Therapy as Salvage Treatment for Advanced Biliary Tract Cancer: A Comprehensive Meta-Analysis of Published Data. Crit. Rev. Oncol. Hematol..

[30] Fornaro L., Vivaldi C., Cereda S., Leone F., Aprile G., Lonardi S., Silvestris N., Santini D., Milella M., Caparello C. (2015). Second-Line Chemotherapy in Advanced Biliary Cancer Progressed to First-Line Platinum-Gemcitabine Combination: A Multicenter Survey and Pooled Analysis with Published Data. J. Exp. Clin. Cancer Res..

[31] Neuzillet C., Casadei Gardini A., Brieau B., Vivaldi C., Smolenschi C., Brandi G., Tougeron D., Filippi R., Vienot A., Silvestris N. (2019). Prediction of Survival with Second-Line Therapy in Biliary Tract Cancer: Actualisation of the AGEO CT2BIL Cohort and European Multicentre Validations. Eur. J. Cancer.

[32] Brieau B., Dahan L., De Rycke Y., Boussaha T., Vasseur P., Tougeron D., Lecomte T., Coriat R., Bachet J.B., Claudez P. (2015). Second-Line Chemotherapy for Advanced Biliary Tract Cancer after Failure of the Gemcitabine-Platinum Combination: A Large Multicenter Study by the Association Des Gastro-Entérologues Oncologues. Cancer.

[33] Abou-Alfa G.K., Macarulla T., Javle M.M., Kelley R.K., Lubner S.J., Adeva J., Cleary J.M., Catenacci D.V., Borad M.J., Bridgewater J. (2020). Ivosidenib in IDH1-Mutant, Chemotherapy-Refractory Cholangiocarcinoma (ClarIDHy): A Multicentre, Randomised, Double-Blind, Placebo-Controlled, Phase 3 Study. Lancet Oncol..

[34] Javle M., Kelley R.K., Roychowdhury S., Weiss K.H., Abou-Alfa G.K., Macarulla T., Sadeghi S., Waldschmidt D., Zhu A.X., Goyal L. (2018). Updated Results from a Phase II Study of Infigratinib (BGJ398), a Selective Pan-FGFR Kinase Inhibitor, in Patients with Previously Treated Advanced Cholangiocarcinoma Containing FGFR2 Fusions. Ann. Oncol..

[35] Doebele R.C., Drilon A., Paz-Ares L., Siena S., Shaw A.T., Farago A.F., Blakely C.M., Seto T., Cho B.C., Tosi D. (2020). Entrectinib in Patients with Advanced or Metastatic NTRK Fusion-Positive Solid Tumours: Integrated Analysis of Three Phase 1–2 Trials. Lancet Oncol..

[36] Hong D.S., Bauer T.M., Lee J.J., Dowlati A., Brose M.S., Farago A.F., Taylor M., Shaw A.T., Montez S., Meric-Bernstam F. (2019). Larotrectinib in Adult Patients with Solid Tumours: A Multi-Centre, Open-Label, Phase i Dose-Escalation Study. Ann. Oncol..

[37] Ramjeesingh R., Chaudhury P., Tam V.C., Roberge D., Lim H.J., Knox J.J., Asselah J., Doucette S., Chhiber N., Goodwin R. (2023). A Practical Guide for the Systemic Treatment of Biliary Tract Cancer in Canada. Curr. Oncol..

[38] Tam V.C., Ramjeesingh R., Burkes R., Yoshida E.M., Doucette S., Lim H.J. (2022). Emerging Systemic Therapies in Advanced Unresectable Biliary Tract Cancer: Review and Canadian Perspective †. Curr. Oncol..

